# Emerging roles of frailty and inflammaging in risk assessment of age-related chronic diseases in older adults: the intersection between aging biology and personalized medicine

**DOI:** 10.7603/s40681-015-0001-1

**Published:** 2015-02-02

**Authors:** I-Chien Wu, Cheng-Chieh Lin, Chao A. Hsiung

**Affiliations:** 1 Institute of Population Health Sciences, National Health Research Institutes, No. 35 Keyan Road, Zhunan, Miaoli County 350, Miaoli, Taiwan; 2 Program for Ageing, College of Medicine, China Medical University, Taichung 404, 404 Taichung, Taiwan; 3 Department of Family Medicine, China Medical University Hospital, 404 Taichung, Taiwan

**Keywords:** Aged;, Aging physiology;, Biological markers;, Chronic disease;, Delivery of health care;, Frail elderly;, Gait;, Geriatric assessment;, Humans;, Health services for the aged;, Inflammation;, Personalized medicine;, Prognosis;, Risk factors;, Risk assessment;, Treatment outcome

## Abstract

A chronic disease in older adults usually runs a course that is less predictable than in younger individuals. Unexplained variations in disease incidence, prognosis, therapeutic responses, and toxicity are frequently observed among older adults. This heterogeneity poses huge challenges to the current one-size-fits-all health care systems, and calls for more personalized managements of chronic diseases in older adults. Aging is characterized by progressive deterioration of bodily functions with increasing risk of failure over time. The entire process is hierarchically organized, and progresses from intracellular events to changes at systemic and ultimately organism levels at different rates among different individuals. Aging biology exerts great influences on the development and progression of most age-related chronic diseases. Thus, aging biology could contribute to the complexity of illnesses that increase with age, and aging biomarkers possess a great potential to enable personalized health risk assessment and health care. We review evidences supporting the roles of aging biomarkers in risk assessment of prevalent age-related diseases. Frailty phenotype is an objectively measured indicator of advanced-stage aging that is characterized by organism-level dysfunction. In contrast, altered inflammation markers level signifies an earlier stage between cellular abnormalities and systems dysfunction. Results of human observational studies and randomized controlled trials indicate that these measures, albeit simple, greatly facilitate classification of older patients with cancer, chronic kidney disease, cardiovascular diseases and type 2 diabetes mellitus into groups that vary in disease incidence, prognosis and therapeutic response/toxicity. As the detailed mechanisms underlying the complex biologic process of aging are unraveled in the future, a larger array of biomarkers that correlate with biologic aging at different stages will be discovered. Following the translational research framework described in this article, these research efforts would result in innovations in disease prevention and management that address the huge unmet health needs of aging populations.

## 1. Introduction

Population aging is prevalent worldwide, and the number of older adults is increasing at an accelerating rate [1]. It is estimated that by 2050, among many nations worldwide at least 20% of the national population will be aged ≥ 60 years [1]. Of note, the most substantial increase has been observed in the oldest-old group (aged > 85 y). Population aging occurs at various rates in different geographic regions. Although at present, Europe contains the most aged population, it is anticipated that by 2050, Asia, South America, and Africa will experience the most rapid rate of increase in population aging.

These demographic changes exert substantial growing pressure on health care in many countries worldwide [2]. A paradigm shift is urgently required in the care for complex chronic diseases and disabilities [2]. Concurrently, we are standing at the dawn of a profound change in our understanding of the aging process, which represents the primary biological underpinning of most chronic diseases and late-life disabilities [3]. Armed with these advances, we have the unique opportunity to address these grand challenges by designing and implementing an effective and sustainable health care system for aging populations.

**Fig. 1:**
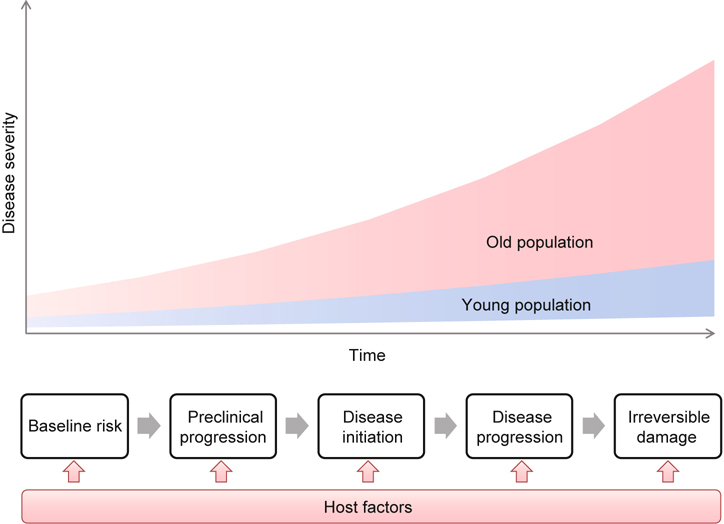
Model of Disease Development. Environmental exposure plus host’s susceptibility (baseline risk) initiates the disease development process, which progresses from the preclinical to the clinical stage and ultimately the irreversible stage [102]. However, as organisms age, an increasing number of host factors could potentially interact the process at any stage, leading to unforeseen heterogeneity in the disease development that could not be explained by this model.

## 2. Challenges of health care in an aging population

Health care for older people differs from that for younger adults and presents great and unique challenges. Older adults are highly susceptible to diseases and disabilities [4]. The prevalence of many chronic diseases, including cardiovascular diseases, cancers, diabetes and respiratory diseases, is high in the elderly population and continues to increase [4-6]. Moreover, older adults tend to have multiple coexisting health conditions. The prevalence of multimorbidity, defined as the coexistence of ≥ 2 chronic diseases or conditions, increases considerably with age and is projected to increase further [7, 8]. Individuals with multiple coexisting health conditions exhibit different and more complex health care needs compared with those with a single health condition [9-11]. In addition, adults with multiple health conditions exhibit considerable differences regarding their health status and health care needs [9-12]. This heterogeneity in the health status and health care needs among older adults challenges the current one-size-fits-all health care systems based on the single-disease paradigm [7, 13-15].

This heterogeneity in health status increases with age and is predominantly observed in older adults (Figure 1) [7, 9, 16]. This heterogeneity is largely attributed to the complex interactions among host factors and disease biology [17-19]. During the course of a chronic disease, numerous host factors, including comorbid age-related conditions, could have profound effects on disease development and progression, thereby altering disease risk, health outcomes, and responses to interventions. These alterations often cause unforeseen variations in disease incidence, prognosis, therapeutic responses, and toxicity among older adults. With the lack of complete consideration of these host factors, traditional single disease paradigm fails to completely explain, predict, and manage these variations [7, 13-15]. Examples are discussed in this section to explain this concept.

Cancer is prevalent in older adults [20]. Over half of new cancers occur in the elderly population [20, 21]. However, despite rapid progress in this field, how to deliver optimal care for older adults with cancer remains unclear [14, 15, 19, 22]. Complex interactions between host biology and cancer biology could result in unpredictable variations in cancer progression, prognosis, treatment responses, and toxicities in an individual. For instance, many chronic illnesses afflicting older adults could affect the overall survival and prognosis of most types of cancer [23, 24]. Studies have even indicated that comorbid illnesses prevalent in older patients with cancer could directly affect the cancer biology [25-27]. In addition, accumulating evidence suggests that chronic diseases may enhance the toxicity of chemotherapy and alter treatment responses [28-30]. The resulting heterogeneity in patients with cancer precludes the appropriate extrapolation of clinical trial results derived from younger or more selective older populations [14, 15, 19, 22].

Similarly, the management of cardiovascular diseases in older adults is complicated by the heterogeneity in the health status of each individual patient. Various chronic diseases or conditions are prevalent in older adults with cardiovascular disease and could considerably affect the disease progression and prognosis through unclear mechanisms [7, 24, 31-35]. For instance, studies have reported that coexisting chronic kidney disease greatly increases the risk of cardiovascular morbidity and mortality [31-33]. Diabetes and myriads of related metabolic disorders not only increase the risk of developing cardiovascular disease for older adults but also accelerate the progression of vascular pathologies [24, 34, 36-38]. Indeed, evidence indicates bidirectional or even more complicated relationships among these chronic diseases with each disease increasing the risk for the other and resulting in a poorer prognosis than with either disease alone [39]. Furthermore, the benefits and harms of treatment of one disease could considerably vary among older patients with different profiles of comorbid conditions [12, 39-41]. The detailed mechanism is presently a subject of intensive research. Without considering these complex interactions and the resulting variations, it is likely that an older individual at a high risk of developing cardiovascular disease would not be targeted for preventive measures (or vice versa), assigned a prognosis that is questionable after developing the disease, and provided an intervention to which the patient responds poorly or with unintended side effects [42, 43].

**Fig. 2 –:**
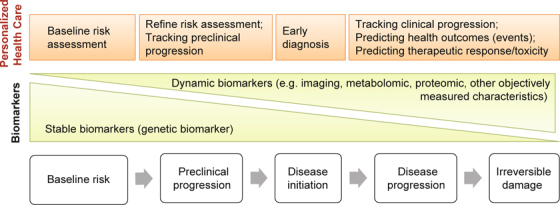
Central roles of biomarkers in personalized medicine. Biomarkers that indicate the activities of diseases pathogenesis at each stage could enhance baseline risk assessment, tracking of preclinical and clinical progression, prediction of health outcomes, therapeutic response or toxicity, thereby enabling personalized disease screening, prevention, diagnosis, prognosis assignment, and therapeutic decisions. Different biomarkers may play distinct roles.

To provide optimal care for older adults, breakthrough innovations are required in disease prevention, diagnosis, and management, based on the uniqueness of individual patients in the complex interaction among host factors and disease biology.

## 3. Personalized medicine

Personalized medicine is defined as tailoring health care based on the individual characteristics of each patient [44]. This is achieved by classifying individuals into groups that exhibit varied disease susceptibility, disease activity, or treatment responses, which thus enables focusing preventive or therapeutic interventions on those who will benefit and sparing those who will not [44]. This classification is based on health risk assessment and relies on the detailed and complete understanding of healthy and diseased states. With recent rapid advances in the understanding of the molecular underpinnings of these states, it is possible to design individualized health care plans based on a person’s unique attributes [17, 19, 22, 42, 44].

Personalized health risk assessment is the core of personalized health care. Traditionally, health risk is assessed using clinical, demographic, and laboratory risk factors; examples include the well-known “factors of risk” obtained from the Framingham coronary heart disease model [45]. This approach remains essentially unaltered and is still widely used in risk prediction. Although these risk factors could be routinely collected in a cost-effective way during clinical practice, their values in personalized health care have been limited by their relatively low accuracy in predicting health outcomes [46-49]. Compared with the traditional risk factors, an individual’s characteristics that are directly related to the diseases pathogenesis could provide more insights into the biologic process, starting from baseline disease susceptibility to disease progression and therapeutic response, thereby allowing more accurate and individualized health risk assessment (Figure 2) [17, 19, 50].

These characteristics that could be objectively measured and evaluated as indicators of normal biological processes, pathogenic processes, or pharmacological responses to a therapeutic intervention of an individual are commonly known as biomarkers and are considered the core of personalized health care [44, 50-53]. Most recent efforts in realizing personalized medicine have largely focused on the use of an individual’s genomic information [17-19, 44, 50, 54]. However, any biomarker could potentially enable the following key tasks in personalized medicine: baseline risk assessment, tracking preclinical and clinical progression, predicting health outcomes, therapeutic responses, and toxicity [44, 50, 52, 53].

Different biomarkers may play distinct roles in personalized disease screening, diagnosis, prognosis assignment, and therapeutic decisions. For instance, a genetic biomarker that indicates an upstream biologic process in the causal pathway of a disease may enhance baseline risk assessment, prediction of therapeutic response, and health outcomes, thereby contributing to personalized diseases screening, diseases prevention, therapeutic decisions, and prognosis assignment [17-19, 44, 50, 54]. A dynamic biomarker, which indicates ongoing biological activities (e.g. imaging markers, metabolomic markers, proteomic markers, and other objectively measured characteristics) is informative in tracking preclinical and clinical disease progression, early diagnosis, and predicting associated health outcomes [44, 50, 53].

Translating biomarkers discoveries into clinical applications typically requires 4 phases of research [55]. The first research (T1) phase examines the candidate applications of a biomarker in screening, diagnosis, prognosis, or therapeutic decisions by, for instance, determining the association of the biomarker with a health outcome in human observational studies. The second phase (T2) assesses the clinical utility of the biomarker, and evidence- based guidelines are established. The third (T3) and fourth (T4) phases examine the dissemination of the practice guidelines into clinical practice and its effects on human health, respectively.

**Fig. 3:**
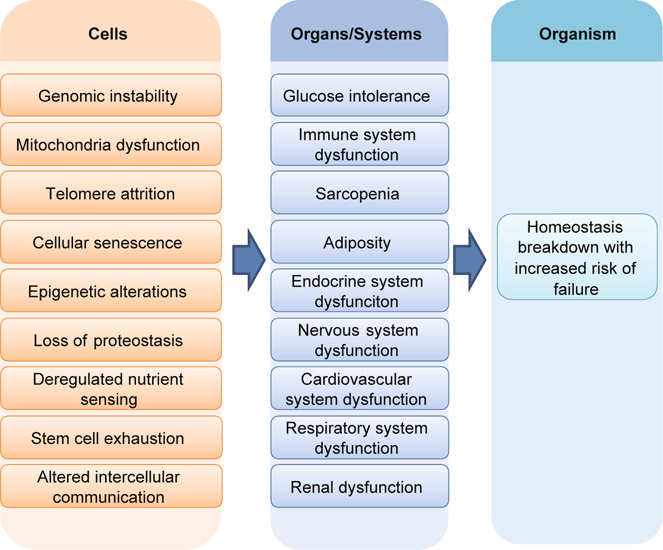
A hierarchical aging model. Cellular abnormalities, including genomic instability, telomere attrition, epigenetic alterations, loss of proteostasis, deregulated nutrient sensing, mitochondrial dysfunction, cellular senescence, stem cell exhaustion, and altered intercellular communication, lead to dysfunction of physiological systems, which, once reaching a threshold, causes organism-level dysfunction characterized by an increased risk of failure (e.g. death and disability).

## 4. Translating aging biology to individualized health care for older people with complex health care needs

By using biomarkers indicating host biology that profoundly affects disease pathogenesis, personalized health risk assessment would facilitate classification of heterogeneous older patients into groups that vary in disease incidence, progression, prognosis, therapeutic response, and toxicity and set the stage for individually tailored disease prevention, diagnosis, and management for each older patient. It is increasingly recognized that biology of aging exerts great influences on the pathogenesis of most chronic diseases prevalent in older adults [3, 56-60]. Thus, an individual’s characteristics that can be objectively measured and evaluated as an indicator of aging biology may serve as a universal biomarker with wide applications and great potential to renovate health care in aging population.

### 4.1. Aging biology

Aging is characterized by progressive deterioration of bodily functions with increasing risk of failure over time [61, 62]. This time-dependent phenomenon typically results from the accumulation of damage over the lifetime, which could differ among different individuals [63]. Decades of research and recent rapid progress has improved the understanding of the possible causes of this damage, the detailed nature of the damage, the processes through which the damage accumulates and leads to progressive deterioration of bodily function and increases the risk of failure [3, 63]. The entire process is hierarchically organized, beginning from intracellular events and followed by changes at cellular, systemic, and ultimately organism levels (Figure 3). The intracellular and cellular events that contribute to aging include genomic instability, telomere attrition, epigenetic alterations, loss of proteostasis, deregulated nutrient sensing, mitochondrial dysfunction, cellular senescence, stem cell exhaustion, and altered intercellular communication (Figure 3) [3]. The consequent damage can alter cell population or function, thereby leading to dysfunctioning of physiological systems (e.g. immune system, musculoskeletal system, cardiovascular system, endocrine system, and neurologic systems) (Figure 3). The survival and health of an organism relies on the dynamic interactions among multiple physiological systems, which allow the organism to mount an appropriate response when challenged by stress, thereby maintaining homeostasis. Reduced functioning of multiple systems could degrade these dynamic interactions, consequently impairing the mechanisms responsible for maintaining homeostasis and increasing the risk of failure at the organism level (e.g. onset and progression of clinical diseases, death, and disability) in the presence of stress [3, 63].

As a result of the detailed elucidation of aging biology, numerous biomarkers that are associated with aging biology at various stages are being discovered. Moreover, emerging evidence indicates that these biomarkers of aging could revolutionize health care by facilitating personalized health risk assessment and individualized health care for older people. Examples will be provided below. We focus on biomarkers which are progressing through the translational research phases described previously and may have potential use in health care in the near future.

**Table 1:** Frailty phenotype according to Fried *et al*. [67]^a^.

Criteria	Frailty Characteristics	Measure
1	Weight loss (unintentional) Shrinking Sarcopenia	>10 lbs lost unintentionally in prior year (reported)
2	Muscle weakness	Grip strength below cutoff value, [67] adjusted for sex and body mass index
3	Exhaustion Poor endurance	Answering “moderate or most of the time” to “I feel that everything I do is an effort” and “I cannot get going.”
4	Slow walking speed	Walking speed below cutoff value, [67] based on the time to walk 15 feet, adjusting for sex and standing height.
5	Low physical activity	Kilocalories expended per week (< 383 kcal/wk in men and < 270 kcal/wk in women)

^a^An individual is considered frail when ≥ 3 of the 5 criteria are present. People with one or 2 of the criteria are considered prefrail, whereas those without any criteria are considered robust (adapted from Wu *et al*. [66]).

### 4.2. Frailty phenotype and health risk assessment

As described previously, once aging progresses to advanced stages involving organism-level dysfunction, it could precipitate organismlevel failure when challenged by stress and lead to clinical disease initiation or progression, disability, and death. Therefore, biomarkers indicating this stage of aging could be applied to predict morbidity, mortality, disability, and other adverse health outcomes in older adults and classify older patients into groups with varying disease risk, prognosis, and therapeutic response or toxicity, thereby setting the stage for customized disease management for each older patient.

An example of this class of biomarker is frailty, which is an objectively measured indicator of aging reaching the organism level [64-66]. As expected, it is characterized by increased vulnerability and a decreased ability to maintain homeostasis [64-66]. In addition, this vulnerability is caused by reduced reserve capacity of the interconnected physiological systems that adapt to stressors, leading to an increased risk of homeostasis failure [64-66]. According to the original operational definition of the frailty phenotype proposed by Fried et al, a person is considered frail when ≥ 3 of the following 5 criteria are present: unintentional weight loss, muscle weakness, slow walking speed, low physical activity, or exhaustion (Table 1) [67]. Older adults with one or 2 of the criteria are considered prefrail, whereas those without any criteria are considered robust [67].

Accumulating evidence supports the utility of frailty phenotype in personalized health risk assessment for older people. Table 2 outlines these studies. Prospective observational studies have repeatedly revealed that frailty predicts multiple adverse health outcomes, including disabilities and death [67-70]. In addition, a higher degree of frailty is associated with greater risks. Studies have further demonstrated that frailty could be applied for risk stratification among patients with chronic diseases. In a recent study, Ness et al observed that patients with cancer who were frail were more likely to die as compared to those who were not frail [71]. Among 1576 incident patients receiving maintenance dialysis, Bao et al observed that frailty was associated with a high risk of future hospitalization and death [72]. Emerging evidence has suggested that frailty phenotype may predict incident clinical diseases[71], although further research is required in this area.

As a simple objective characteristic, gait speed is considered a major component of the frailty phenotype. Similar to frailty, slow gait speed alone has been shown to be a strong and independent predictor of numerous major health outcomes in older adults [73, 74]. Older adults with slow gait speed are at a high risk of future disability [74, 75]. A pooled analysis of 9 major cohort studies demonstrated that a slow gait speed predicted mortality in older people [76]. Adult patients with chronic kidney disease who walked slower were at a higher risk of death [77]. Dumurgier et al observed that older adults with slow walking speed exhibited an approximately 3-fold higher risk of cardiovascular death [78]. Roles of gait speed in risk stratification among patients with cardiovascular diseases have been further supported by recent studies [79, 80]. In particular, Afilalo et al reported that older patients with slow walking speed were more likely to experience complications, including postoperative mortality and major morbidity, following cardiac surgery [80]. Older adults with slow gait speed were at higher risk of developing clinical diseases in the future [81].

The clinical utility of frailty phenotype was recently examined in a randomized controlled trial, wherein the authors investigated whether frailty assessment has the potential to change the management of complex chronic diseases prevalent in older adults and improve outcomes [82]. Treatment of type 2 diabetes mellitus in older adults is challenging. Both inadequate treatment and treatment complications (e.g. hypoglycemia) are major concerns that are frequently encountered among older patients [83]. Using a randomized controlled trial, Strain et al demonstrated that drug treatment guided by frailty assessment is feasible. In addition, this individualized care could not only facilitate adequate treatment but also minimize the risk of treatment complications [82].

### 4.3. Inflammation markers and health risk assessment

Inflammation causes aging and represents a crucial cellular-level process that could lead to dysfunction of multiple physiological systems and subsequent organism-level dysfunction [3, 84]. Inflammation not only accelerates aging but also plays a major role in the pathogenesis and progression of age-related diseases [85-87]. For instance, a recent large-scale human genetic study suggested that interleukin-6 (IL-6) signaling pathway is causally related to coronary heart disease [88]. The biology of aging exerts major influences on the pathogenesis of chronic diseases through inflammation [3, 84].

Inflammation associated with aging is often determined by measuring the levels of cytokines, including IL-6, interleukin-1β (IL-1β) and tumor necrosis factor-α (TNF-α), in the blood [84-89]. Acute phase proteins (e.g. C-reactive protein), which are produced by liver cells in response to gp140-meditated transsignaling triggered by IL-6 and easily detected in circulating blood, represent another group of biomarkers frequently used to assess inflammation of aging [90].

**Table 2:** Studies supporting the roles of frailty in personalized health risk assessment.

Studies	Markers	Design	Population	Outcomes	Key Findings	Translation Phase^a^
Fried *et al*. [67]	Frailty phenotype	Longitudinal study (7 y)	5317 men and women aged ≥ 65 y	Hospitalization, falls, disability, and mortality	Frailty phenotype predicted incident hospitalization, falls, worsening disability, and death.	T1
Bandeen- Roche *et al*. [69]	Frailty phenotype	Longitudinal study (3 y)	1438 women aged ≥ 65 y	Institutionalization, disability, and mortality	Frailty phenotype predicted incident institutionalization, worsening disability, and death.	T1
Ensrud *et al*. [70]	Frailty phenotype	Longitudinal study (4.5 y)	6701 women aged ≥ 69 y	Falls, disability, and mortality	Frailty phenotype predicted incident falls, worsening disability, and death.	T1
Ness *et al*. [71]	Frailty phenotype	Longitudinal study	1922 adult childhood-cancer survivors aged ≥ 18 y	Morbidity and mortality	Frailty phenotype predicted incident morbidity and death.	T1
Bao *et al*. [72]	Frailty phenotype	Longitudinal study (1.2 y)	1576 incident patients receiving maintenance dialysis	Hospitalization and mortality	Frailty phenotype predicted incident hospitalization and death.	T1
Strain *et al*. [82]	Frailty phenotype	Marker-guided randomized control trial (24 wk)	278 patients with type 2 diabetes aged ≥ 70 y	Proportion of patients reaching HbA1c target and HbA1c reduction (Vildagliptin *vs*. placebo)	Frailty-guided drug treatment was effective in achieving HbA1c target and HbA1c reduction without any tolerability concerns.	T2
Studenski *et al*. [76]	Gait speed	Longitudinal study (6-15 y)	34 485 men and women aged ≥ 65 y	Mortality	Slower gait speed was associated with higher risk of death.	T1
Dumurgier *et al*. [78]	Gait speed	Longitudinal study	3208 men and women aged ≥ 65 y	Mortality	Slower gait speed predicted incident cardiovascular death.	T1
Chaudhry *et al*. [79]	Gait speed	Longitudinal study (3.4 y)	758 men and women aged ≥ 65 y with incident heart failure.	Hospitalization	Gait speed less than 0.8 m/s predicted incident hospitalization.	T1
Afilalo *et al*. [80]	Gait speed	Longitudinal study (5.2 y)	131 men and women aged ≥ 70 y receiving cardiac surgery	Inpatient postoperative mortality and major morbidity	Gait speed less than 0.8 m/s predicted inpatient postoperative mortality and major morbidity.	T1
Roshanravan *et al*. [77]	Gait speed	Longitudinal study (3 y)	385 adult patient aged > 18 y with chronic kidney disease	Mortality	Slower gait speed was associated with higher risk of death.	T1
McGinn *et al*. [81]	Gait speed	Longitudinal study (5.2 y)	13048 women aged ≥ 65 y	Incident ischemic stroke	Slower gait speed was associated with higher risk of incident ischemic stroke.	T1

^a^Refer the text for detailed description.

Studies have revealed that these inflammatory biomarkers could be useful in predicting morbidity, mortality, disability, and multiple adverse health outcomes (Table 3). High inflammatory marker levels are associated with higher risks of multiple adverse health outcomes, including falls, disabilities, and death among older adults [91-94]. Moreover, studies have suggested that inflammatory markers may aid in risk stratification among patients with chronic diseases. For instance, Volpato et al observed that increased serum IL-6 levels predicted death among older women with cardiovascular disease [95]. Another study revealed that among patients receiving isolated elective coronary artery bypass grafting, those with an IL-6 gene promoter variant associated with high postoperative IL-6 levels were more likely to experience postoperative atrial fibrillation as a major postoperative complication [96]. Furthermore, as an indicator of upstream intracellular and cellular events that cause aging, these inflammatory markers predicted the incidence of multiple age-related chronic diseases [97-101].

**Table 3:** Studies supporting the roles of inflammatory markers in personalized health risk assessment.

Studies	Markers	Design	Population	Outcomes	Key Findings	Translation Phase^a^
Akbaraly *et al*. [91]	IL-6	Longitudinal study (10 y)	3044 men and women aged ≥ 49 y	Morbidity and mortality	High levels of IL-6 predicted incident cardiovascular disease and death.	T1
Newman *et al*. [92]	IL-6	Longitudinal study (16 y)	5888 men and women aged ≥ 65 y	Mortality	High levels of IL-6 predicted death.	T1
Jenny *et al*. [93]	CRP, fibrinogen	Longitudinal study (5 y)	5828 men and women aged ≥ 65 y	Mortality	High levels of CRP and fibrinogen were more strongly associated with death in older men than women and more strongly associated with early than late death.	T1
Cohen *et al*. [94]	IL-6, D-dimer	Longitudinal study (5 y)	1723 men and women aged ≥ 72 y	Mortality and disability	High levels of IL-6 and D-dimer predicted death and disability	T1
Kalogeropoulos *et al*. [97]	IL-6, TNF-α, CRP	Longitudinal study (9.4 y)	2610 men and women aged ≥ 70 y	Incident heart failure	High levels of IL-6 and TNF-α predicted incident heart failure.	T1
Cesari *et al*. [98]	IL-6, TNF-α CRP	Longitudinal study (3.6 y)	2225 men and women aged ≥ 70 y	Incident coronary heart disease, stroke, and congestive heart failure	High levels of IL-6 and TNF-α predicted incident coronary heart disease, stroke, and congestive heart failure.	T1
Pradhan *et al*. [99]	IL-6, CRP	Prospective, nested case-control study (2.9 y)	608 women aged ≥ 50 y	Incident coronary heart disease	High levels of IL-6 and CRP predicted incident coronary heart disease.	T1
Volpato *et al*. [95]	IL-6	Longitudinal study (3 y)	620 women aged ≥ 65 y	Mortality	High levels of IL-6 predicted death among those with cardiovascular disease.	T1
Pradhan *et al*. [100]	IL-6, CRP	Prospective, nested case-control study (4 y)	550 women aged ≥ 65 y	Incident type 2 diabetes	High levels of IL-6 and CRP predicted incident type 2 diabetes.	T1
Hu *et al*. [101]	IL-6, TNF-α receptor 2, CRP	Prospective, nested case-control study (10 y)	1522 women aged ≥ 43 y	Incident type 2 diabetes	High levels of IL-6, TNF-α receptor 2 and CRP predicted incident type 2 diabetes.	T1

^a^Refer the text for detailed description.

## 5. Conclusion and future perspectives

The biology of aging greatly influences the development and progression of most diseases, disabilities, and other health conditions among older adults. Therefore, detailed elucidation of the aging process would shed new light on the common pathway contributing to complex chronic illnesses developing in later stages of life and facilitate the development of universal biomarkers that can personalize health risk assessment and health care in older patients with a wide range of health conditions.

Frailty phenotype and inflammatory markers show great promise in this regard. However, further investigations (T2, T3, and T4 studies) are required to firmly establish their clinical utility in health risk assessment. Concurrently, with increasing knowledge regarding the detailed aging process, novel biomarkers that correlate with biologic aging at different stages can be discovered. Similarly, their potential roles in personalized health risk assessments in older patients should also be critically examined in human studies, beginning from the T1 phase of translational research. These efforts will ultimately unleash a renovation of health care that meets the needs of the increasingly aged population worldwide.
